# “Not enough” vs. “never perfect”: a qualitative analysis of penile enlargement surgery intentions in heterosexual and homosexual men

**DOI:** 10.1093/sexmed/qfag024

**Published:** 2026-05-15

**Authors:** Boqiang Zhang, Tie Li, Bin Lei

**Affiliations:** The First Affiliated Hospital, Jinan University, Guangzhou, 510632, China; The First Affiliated Hospital, Jinan University, Guangzhou, 510632, China; The First Affiliated Hospital, Jinan University, Guangzhou, 510632, China

**Keywords:** penile enlargement, sexual orientation, body image, masculinity, cosmetic surgery, qualitative study

## Abstract

**Background:**

Psychological motivations for penile enlargement surgery (PES) remain understudied, particularly across different sexual orientations.

**Aim:**

This study explores the psychosocial drivers of PES intentions among heterosexual and homosexual cisgender men.

**Methods:**

Twenty-four cisgender men (12 heterosexual, 12 homosexual) who had undergone or recently consulted for PES were interviewed using semi-structured protocols. Data were analyzed via thematic analysis, guided by the Push-Pull Theoretical Model. Push factors refer to internal pressures that motivate individuals to seek PES, while pull factors involve external influences.

**Outcomes:**

Distinct motivational pathways toward PES were identified based on sexual orientation.

**Results:**

Two shared pull factors, physiological safety concerns and social exposure risks, were evident across groups. However, push factors varied: heterosexual men emphasized partner-driven pressures such as sexual performance anxiety, masculine adequacy concerns, and partner-related comparison. Homosexual men highlighted community-driven influences, including body image anxiety, perfectionistic enhancement culture, and peer-based comparison norms. These findings reveal two diverging logics: relational adequacy versus visual optimization.

**Clinical Translation:**

Tailored psychological screening and counseling strategies should consider the influence of sexual orientation and underlying motivational logic.

**Strengths and Limitations:**

This study addresses a gap in male body image and LGBTQ+ surgical intention literature. Limitations include a modest, urban-leaning sample and a focus on intention rather than postoperative outcomes.

**Conclusion:**

Sexual orientation significantly shapes men’s motivations for PES, with heterosexual men driven by partner-related anxiety and homosexual men influenced by community-based ideals. Understanding these distinctions is critical for ethical and effective clinical intervention.

## Introduction

Genital cosmetic surgery research has largely focused on women, particularly procedures like labiaplasty,^1^^,^^2^ leaving male genital surgeries such as penile enlargement surgery (PES) underexplored.^3^^,^^4^ Existing PES literature is mostly clinical, discussing methods, outcomes, or complications,^5–7^ while psychological and sociocultural motivations remain understudied.^8^^,^^9^ Moreover, most studies treat men as a homogenous group, overlooking differences based on sexual orientation.^10^^,^^11^

Emerging research suggests that heterosexual and homosexual men may experience genital dissatisfaction differently.^12^^,^^13^ Heterosexual men often express anxiety over sexual performance and partner satisfaction,^14^ while homosexual men may be influenced more by visual culture, peer comparisons, and aesthetic ideals, especially within dating and community contexts.^15^ These distinctions, however, have seldom been applied to motivations for PES. This study aims to address this gap by exploring the psychological drivers behind PES intentions in both heterosexual and homosexual cisgender men. We propose that motivations for PES differ across these two groups, influenced by unique psychosocial factors shaped by sexual orientation.

Based on the Push-Pull Theoretical Model^16^^,^^17^ and qualitative interviews and thematic analysis,^18^^,^^19^ this study conceptualizes push factors as internal pressures (eg, body dissatisfaction, performance anxiety) that motivate individuals to pursue PES and pull factors as external influences (eg, societal expectations, community standards) that make the procedure appear more attractive. By investigating these motivational pathways, we aim to contribute to the understanding of how masculinity, body image, and sexual orientation intersect in shaping male cosmetic surgery decisions. We hypothesize that heterosexual men are primarily driven by relational inadequacy, while homosexual men are more influenced by community-driven ideals and visual optimization.

## Methods

### Design

This study used a qualitative design based on constructivist grounded theory to explore men’s motivations for considering PES. Given the sensitive, identity-related nature of the topic, semi-structured, in-depth interviews were chosen to capture participants’ lived experiences in detail.^20^ The interview guide covered body image, masculinity, sexual performance, and social influence, allowing themes to emerge organically. Participants included heterosexual and homosexual cisgender men, enabling comparison across sexual orientations.^13^^,^^14^ Recruitment was conducted via online forums and social networks.

### Participants and settings

Participants were recruited through the social media platform Red Note, which hosts anonymous health and lifestyle discussion forums frequented by individuals exploring body-related procedures. A digital call-for-participation was posted in male body enhancement and cosmetic surgery subgroups, detailing the study’s focus on the psychological and social motivations for considering or undergoing PES.

To ensure the relevance and authenticity of participant experiences, strict inclusion criteria were applied. Eligible participants met at least one of the following three conditions: (1) Had visited a hospital or private clinic to consult about PES within the past 3 months, with verifiable medical documentation (eg, outpatient consultation records or chat transcripts with licensed providers); (2) Had undergone any form of penile enlargement surgery, including injectable fillers, ligament release, or implant-based procedures; (3) Had seriously planned PES in the last year, including conducting medical research, scheduling consultations, or saving for surgical costs.

Participants were also required to be >18 years old, identify as cisgender men, and self-report either a heterosexual or homosexual sexual orientation. Initial screening was conducted through private messaging and a short online pre-interview form. Medical consultation proof was submitted securely and reviewed with confidentiality.

After this filtering process, 24 participants were included in the final sample, with ages ranging from 21 to 45 years. Of these, 9 participants met the first inclusion criterion (consulting about PES at a hospital or private clinic), 9 participants met the second criterion (having undergone penile enlargement surgery), and 15 participants met the third criterion (having seriously planned PES within the past year). Data collection took place between April and August 2025. Participants received modest compensation for their time.

### Procedures

Each participant completed a 60-90-min semi-structured interview via a secure online platform. The interviews were conducted by B.Z., with assistance from T.L. and B.L. The interviews were guided by five key domains: (1) participants’ understanding of penile enlargement surgery (PES) and their motivations for considering it (eg, “What do you understand about penile enlargement surgery?”); (2) their self-perceptions regarding body image and sexual performance (eg, “How do you feel about your body image, particularly regarding your genital area?”); (3) views on partner and societal expectations about masculinity and genital adequacy (eg, “How do you think your partner views your body and genital size?”); (4) perceived risks and barriers—both medical and social (eg, “What are your main concerns about the safety or side effects of PES?”); and (5) emotional responses such as anxiety, doubt, or anticipation throughout the decision-making process (eg, “How did you feel when first considering PES?”). While these domains ensured consistency, interviewers maintained a conversational, adaptive tone, allowing participants to elaborate freely and introduce new or unexpected themes. All interviews were recorded, transcribed, anonymized, and supplemented with post-interview field notes to capture nonverbal cues.^21^

### Data analysis

Data were analyzed using a three-stage thematic coding process grounded in constructivist grounded theory.^22^ First, open coding identified key concepts from participant narratives. Second, axial coding grouped related ideas into subthemes, with comparisons made across sexual orientations. Finally, selective coding integrated these into four major themes aligned with the Push-Pull Model. The team ensured rigor through double coding, team discussions, and saturation checks to confirm thematic completeness.

### Trustworthiness and rigor

The study ensured credibility through double coding, team discussions, and participant feedback. Dependability was maintained via detailed documentation and coding audits, while theoretical saturation confirmed theme completeness.^18–20^ Transferability was supported by rich participant quotes, and confirmability was upheld through reflexivity and external peer review. Ethical standards and data security were strictly followed throughout the research.^19^

## Results

**Figure 1:**
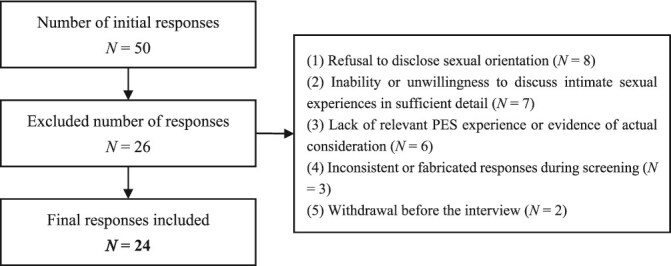
Participant screening flowchart.

As shown in Figure 1, a total of 50 individuals initially responded and expressed willingness to participate. However, 26 cases were excluded after screening for the following reasons: (1) Refusal to disclose sexual orientation (n = 8); (2) Inability or unwillingness to discuss intimate sexual experiences in sufficient detail (n = 7); (3) Lack of relevant PES experience or evidence of actual consideration (n = 6); (4) Inconsistent or fabricated responses during screening (n = 3); (5) Withdrawal before the interview (n = 2).

As shown in Table 1, the final sample included 24 cisgender men evenly split between heterosexual and homosexual orientations, with an average age of 30.7 years (*M*_heter_ = 31.2, *M*_homo_ = 30.2). Most participants were highly educated, with the majority holding bachelor’s or advanced degrees. Occupations were diverse, ranging from professionals and students to freelancers and healthcare workers. Marital status varied, with most participants being single or in a relationship.

**Table 1:** Participants’ characteristics (N = 24).

ID	Age	Sexual orientation	Occupation	Marital status	Education	Undergone PES
P1	40	Heterosexual	IT Consultant	Divorced	Bachelor’s degree	Yes
P2	22	Heterosexual	Student	Single	Bachelor’s degree	No
P3	29	Heterosexual	Marketing specialist	Single	Bachelor’s degree	No
P4	28	Heterosexual	Doctor	Single	PhD	No
P5	43	Heterosexual	Engineer	Divorced	PhD	Yes
P6	41	Heterosexual	Marketing specialist	Married	Master’s degree	Yes
P7	25	Heterosexual	Student	Single	Bachelor’s degree	No
P8	30	Heterosexual	Teacher	Married	Master’s degree	Yes
P9	21	Heterosexual	Student	Single	Bachelor’s degree	No
P10	27	Heterosexual	Worker	In a relationship	High school diploma	Yes
P11	23	Homosexual	Freelancer	In a relationship	Master’s degree	No
P12	30	Homosexual	Marketing specialist	In a relationship	Master’s degree	No
P13	32	Homosexual	Doctor	Single	PhD	Yes
P14	31	Homosexual	Refused to disclose	Single	PhD	Yes
P15	32	Homosexual	Teacher	Single	PhD	Yes
P16	22	Homosexual	Influencers	Single	High school diploma	No
P17	28	Homosexual	Marketing specialist	Divorced	Master’s degree	No
P18	31	Homosexual	IT Consultant	In a relationship	PhD	No
P19	32	Homosexual	Nurse	In a relationship	Bachelor’s degree	No
P20	25	Homosexual	Start a business	Single	Master’s degree	Yes
P21	45	Homosexual	Refused to disclose	Married	Master’s degree	No
P22	42	Heterosexual	Worker	Divorced	Associate degree	No
P23	31	Homosexual	Designer	Single	Master’s degree	No
P24	26	Heterosexual	IT Consultant	In a relationship	Master’s degree	No

Note: Single refers to individuals who are unmarried and not in a relationship, while divorced specifically refers to individuals who were previously married and are now separated. PES, penile enlargement surgery.

## Themes

Table 2 presents the thematic findings based on the Push-Pull Theoretical Model. Across both groups, participants described shared pull factors, primarily concerns over physiological safety (eg, fear of complications) and social risk (eg, fear of being exposed or judged), that influenced the feasibility of undergoing PES.

**Table 2:** Themes and subthemes of this study.

Theme	Subtheme	Illustrative quote
Physiological safety risk	Fear of complications	“If the filler migrates or gets infected, you can be worse off than before. That really scares me.” (P2)
	Lack of long-term evidence	“I feel like no one really talks about the side effects seriously, and the doctors just want your money.” (P6)
	Uncertainty about reversibility	“I don’t mind spending money, but only if it’s tested, proven, and reversible.” (P17)
Social exposure risk	Shame and secrecy	“This is not something you can tell your friends or even your partner. If they find out, it’s humiliating.” (P3)
	Masculinity threat	“Even if it worked, I don’t want her to think I needed it. That would just prove I wasn’t enough.” (P6)
	Fear of gossip	“What if it leaks out somehow? People gossip. That’s the worst part.” (P5)
Partner-driven pressure (heterosexual men)	Sexual performance anxiety	“Every time it doesn’t go perfectly, I wonder if it’s because I’m not big enough.” (P7)
	Masculine adequacy norms	“I don’t feel small, but I don’t feel ‘enough’ either. Like I don’t fully measure up.” (P3)
	Partner comparison anxiety	“She once said her ex was really good in bed. She didn’t say size, but I couldn’t stop thinking about it.” (P5)
Community-driven pressure (homosexual men)	Body image anxiety	“Even when I know I’m not small, I still feel like I’m not big enough—compared to everyone online.” (P17)
	Perfectionism and enhancement	“I don’t hate my body, but I think it could be better. Why not upgrade?” (P13)
	Community comparison norms	“You go to the sauna, the beach, even dating apps—it’s always about who’s bigger, leaner, better.” (P11)

While the push factors differed between groups, they each followed a distinct motivational logic: heterosexual men’s motivations were primarily partner driven, rooted in intimate relationship dynamics and performance anxiety, whereas homosexual men’s motivations were largely community driven, shaped by peer comparison, body image ideals, and aesthetic norms within gay subcultures.

### Pull factors

#### Physiological safety risk

A major shared concern among both heterosexual and homosexual participants was the physiological risk associated with PES. While participants expressed varying degrees of interest in PES, most voiced anxiety regarding the potential for physical complications, including nerve damage, erectile dysfunction, filler migration, and post-surgical deformity. These concerns were particularly acute in cases where participants had conducted prior research or had read unregulated information online.

“I read that if the filler migrates or gets infected, you can be worse off than before. That really scares me.” (P2).

“It’s not like getting abs through the gym. If something goes wrong, you’re risking the most sensitive part of your body.” (P11).

These concerns reflect the uncertainty surrounding the long-term outcomes and variability in medical standards of PES practices. Compared to more established cosmetic surgeries, PES is perceived as lacking in procedural transparency, peer-reviewed safety data, and clear clinical guidelines.

“I feel like no one really talks about the side effects seriously, and the doctors just want your money.” (P6).

“There are too many forums, too many mixed opinions—it’s hard to know who to trust.” (P15).

Some participants pointed out that even minimally invasive options, such as hyaluronic acid fillers, did not completely eliminate the risk. Although such methods were more acceptable, the perceived possibility of adverse outcomes still served as a strong deterrent.

“I’d need to be 100% sure it’s safe. Not just now, but in 10 years. What if it affects fertility or something?” (P8).

“I don’t mind spending money, but only if it’s tested, proven, and reversible.” (P17).

This theme illustrates how physiological safety risk functions as a major pull factor, mediating the gap between desire and action. Even among participants with high body dissatisfaction or motivation to undergo PES, concerns about medical safety substantially decreased the likelihood of surgical pursuit.

#### Social exposure risk

In addition to concerns about physiological harm, participants across both sexual orientation groups expressed significant anxiety over social exposure risk—the fear of being discovered or judged by others for having undergone PES. This concern was deeply tied to feelings of shame, secrecy, and masculine vulnerability.

“This is not something you can tell your friends or even your partner. If they find out, it’s humiliating.” (P3).

“I’d rather deal with the insecurity than have someone think I did something fake to fix it.” (P12).

The decision to pursue PES was therefore not only a medical or aesthetic one but also a reputational negotiation, shaped by assumptions about what the surgery might imply to others. Some heterosexual men worried that disclosure would undermine their sexual credibility or masculinity, while some homosexual men feared that being known to have undergone enhancement would make them seem desperate or inauthentic within peer communities.

“Even if it worked, I don’t want her to think I needed it. That would just prove I wasn’t enough.” (P6).

“In our circle, everything is about image, but ironically you can’t admit you had something done.” (P19).

These statements reflect that PES is often associated with deep-seated insecurity or perceived inadequacy, carrying a heavy symbolic load that makes it highly stigmatized and rarely discussed openly. This makes disclosure anxiety a major deterrent, even when participants felt otherwise motivated or dissatisfied.

“What if it leaks out somehow? People gossip. That’s the worst part.” (P5).

“In the gay community, everything is hyper-sexualized, but you still can’t show weakness.” (P14).

As such, the desire to preserve social anonymity became a core condition for considering PES. Many participants emphasized the appeal of “invisible” procedures—those without scars, downtime, or visible indicators of surgical intervention.

“Only if no one can tell. Not even in the locker room.” (P7).

“If I ever did it, it has to be completely private. Like, no one even suspects.” (P16).

This theme underscores that social exposure risk is not simply about being seen, but about being interpreted, judged, or redefined in undesirable ways. The psychological calculus of PES includes not only the hoped-for outcome, but also the fear of altered social identity.

### Push factors in heterosexual men: the partner-driven pathway

#### Sexual performance anxiety

A central push factor among heterosexual men considering PES was sexual performance anxiety. This anxiety was not necessarily rooted in objective dysfunction, but in subjective fear of inadequacy, particularly in relation to partner satisfaction. For many participants, concerns about penis size were deeply tied to doubts about their ability to meet their female partner’s expectations.

“It’s not just about how I feel—it’s about whether she’s satisfied. If I’m not enough, that’s a failure.” (P4).

“Every time it doesn’t go perfectly, I wonder if it’s because I’m not big enough.” (P7).

This performance anxiety was often anticipatory rather than reactive—some participants had never received explicit negative feedback, yet internalized cultural narratives suggesting that size equates to competence.

“She never complained, but I keep thinking: what if she just didn’t say anything?” (P2).

“I feel like every guy is secretly competing. You never know what your partner is comparing you to.” (P8).

The theme of sexual responsibility was also prominent: participants felt that ensuring female satisfaction was part of their role as a man, and any perceived failure in this domain triggered guilt or shame. These feelings were often exacerbated by pornographic standards, online content, or discussions among peers, which created an unattainable benchmark.

“It’s everywhere—porn, forums, jokes about size. You’re supposed to be this perfect performer.” (P6).

“There’s this pressure that if she doesn’t finish, it’s your fault. It messes with your head.” (P1).

These narratives echo findings in body image and masculinity research that link sexual self-worth to genital dimensions, especially within heteronormative relational scripts. In this context, PES is not just about changing the body—it becomes a symbolic act to restore sexual confidence and male identity.

Importantly, participants did not describe PES as a first option, but rather a potential “last resort” when psychological pressure becomes chronic or when doubts are reinforced by relational feedback:

“If nothing changes and I keep feeling this way, I might do it—just to get it out of my head.” (P5).

Thus, sexual performance anxiety functions as a cumulative push factor: it builds over time, fueled by silence, ambiguity, and self-comparison, eventually leading some men to consider PES as a means of regaining perceived control in intimate relationships.

#### Masculine adequacy norms

Beyond sexual performance anxiety, many heterosexual participants framed their desire for PES as a response to deeper concerns about masculine adequacy. In this context, penis size was not merely a functional attribute, but a symbolic marker of manhood, strength, and self-worth. Participants linked the size of their penis to core aspects of masculine identity, including confidence, authority, and status within both romantic and social contexts.

“You grow up hearing that bigger is better. It’s not just about sex—it’s about being a man.” (P9).

“I don’t feel small, but I don’t feel ‘enough’ either. Like I don’t fully measure up.” (P3).

Participants described how genital size is linked to broader cultural ideals of male dominance and physical superiority, with many expressing a background sense of inadequacy, as if falling short of an imagined masculine standard.

“Nobody ever told me I was small. But still, it’s in the back of your mind—what if you’re not man enough?” (P1).

“It’s more about me than anyone else. I just don’t like feeling average.” (P7).

Participants described how male body image is shaped by cultural ideals of masculinity, where strength, size, and control are highly valued. Within this framework, penile size is often seen as a measure of power and gender legitimacy, reinforced by subtle cues in locker-room talk, media, and health discourse.

“Even when guys joke about size, there’s truth in it. It’s how we rank each other, even silently.” (P6).

“I know it sounds stupid, but size just feels like it completes the picture. Like without it, I’m missing something.” (P2).

For some men, this sense of inadequacy persisted even when they intellectually recognized that their concerns were socially constructed rather than medically significant. The emotional impact of perceived “average” size was not always proportional to their objective measurements, but rather to how they imagined themselves being perceived in masculine hierarchies.

“I’ve been told I’m normal. But what is normal? No one wants to be just ‘fine’.” (P10).

As such, PES was viewed by some not as a necessity, but as a means of aligning their physical body with internalized masculine ideals—an attempt to close the gap between how they appear and how they feel they should appear. The pressure was not always relational or sexual, but existential: a striving to be a man in full.

#### Partner-related comparison

A third significant push factor among heterosexual men was the influence of indirect partner comparison, referring to perceived or imagined comparisons with past partners, media figures, or generalized expectations communicated by their female partners. Even in the absence of explicit criticism, participants described a heightened sensitivity to subtle cues, often interpreting neutral or ambiguous comments as signs of inadequacy.

“She once said her ex was really good in bed. She didn’t say size, but I couldn’t stop thinking about it.” (P5).

“You never know what they’ve had before. Even if they say you’re fine, maybe they’re just being polite.” (P2).

Such comparisons were rarely direct, but they triggered a ruminative loop where men repeatedly questioned whether their partners were fully satisfied or merely tolerant. This sense of sexual competition, even when unspoken, shaped their self-image and led to compensatory behaviors.

“I keep thinking: what if she had someone bigger before? I can’t ask, but I imagine it all the time.” (P9).

“She made a joke once about porn stars, and I laughed, but inside I felt awful.” (P4).

Participants also reported feeling scrutinized during sexual activity, even when partners expressed no dissatisfaction. The presence of a romantic or sexual partner created a mirror effect, where men saw themselves through another’s gaze, intensifying their self-monitoring and fueling anxiety.

“It’s not about what I think. It’s about what I think she’s thinking.” (P3).

“Sex used to be fun. Now it’s like a performance review every time.” (P7).

These dynamics often led participants to view PES as a preemptive defense—a way to eliminate the possibility of being judged or compared negatively, even if no such judgment had occurred.

“Maybe she’s happy now, but what if someone better comes along? I want to stay competitive.” (P8).

This theme reflects how individuals may alter their bodies not in response to failure, but in anticipation of it. In this sense, PES becomes a psychological buffer against imagined relational threats, stemming from deep-seated insecurities about comparison and replacement.

### Push factors in homosexual men: the community-driven pathway

#### Body image anxiety

For homosexual men considering PES, a primary push factor was body image anxiety, specifically related to how one’s genital appearance compared to aesthetic ideals within gay male culture. Unlike the heterosexual participants, whose motivations were often tied to functionality and partner feedback, homosexual participants frequently described their motivations as rooted in self-surveillance and visual comparison, both in digital and offline contexts.

“You go on dating apps and every profile looks like a Greek statue. It messes with your head.” (P12).

“You start to think—if I don’t look a certain way, I’m not even in the game.” (P14).

Many participants described feeling trapped in a culture that equated desirability with hyper-idealized body standards, including muscularity, low body fat, and of course, penile size. This aligns with previous studies showing that gay men experience greater appearance-based self-monitoring and body dissatisfaction than their heterosexual counterparts.

“Even when I know I’m not small, I still feel like I’m not big enough—compared to everyone online.” (P17).

“It’s like you’re always being looked at, even before anyone touches you.” (P19).

This constant visibility created a sense of sexual competition and evaluation, where the body became a product on display. Participants reported that these standards were often internalized from a young age, reinforced by media, pornography, and peer interactions, producing chronic dissatisfaction even when objectively unwarranted.

“I’ve measured myself so many times. It never ends. Like, what’s the number that finally makes me okay?” (P13).

“There’s no finish line. You get fit, you get lean, and then the next thing is: Are you big enough?” (P15).

This theme reflects what scholars describe as “appearance-based rejection sensitivity”, where individuals become hyper-aware of potential negative evaluation based on physical features. In the gay male context, this sensitivity is magnified due to the visual-first nature of social and sexual engagement, where physical appearance often precedes deeper connection.

“In our world, people don’t get to know you first. They look, then swipe.” (P11).

For many participants, PES was not about fixing a deficit but “closing the gap” between their real bodies and internalized ideals. The motivation, therefore, was aspirational rather than corrective—driven by the desire to belong, to compete, and to be seen as desirable in a system of visual capital.

#### Perfectionism and enhancement culture

Beyond generalized body image anxiety, many homosexual participants described their interest in PES as part of a broader culture of aesthetic perfectionism and enhancement normalization. In this framework, the pursuit of physical modifications was not framed as fixing a flaw, but as optimizing an already acceptable body to meet ever-evolving internal and community standards.

“I don’t hate my body, but I think it could be better. Why not upgrade?” (P13).

“It’s not that I’m small. I just want to be the best version of myself.” (P17).

This sentiment reflects a shift from deficit-based motivation toward aspirational enhancement, where PES is viewed as part of a continuum of body work, alongside skincare, fitness routines, fillers, and other non-surgical enhancements.

“All my friends do Botox, laser, some even got pec implants. This isn’t that different.” (P15).

“If you’re already investing in your appearance, why stop there?” (P20).

Several participants reported that within their social circles, body modifications—including genital procedures—were increasingly normalized or even expected. In some subcultures, “doing the work” was seen as a signal of self-respect and social competitiveness, reinforcing a kind of enhancement arms race.

“It’s not just about how you look—it’s about showing that you care enough to improve.” (P12).

“Looking good isn’t optional anymore. Everyone’s upgrading.” (P18).

This mindset reflects the idea that constant self-improvement is a marker of value and desirability. In such environments, PES is seen as a natural extension of other enhancement practices, not a radical medical intervention, but a step toward bodily optimization within a competitive sexual market.

“I see it like going to the gym. Some people build muscles. Some adjust other things.” (P16).

Underlying this enhancement culture is a form of internalized perfectionism, in which individuals continually set new benchmarks for what is “good enough.” Even those who objectively fit mainstream ideals often described feeling not quite there yet, reflecting an endless aesthetic chase.

“Perfection is a moving target. Even if I got it done, I’d probably find something else.” (P14).

In this context, PES is less about identity repair and more about visual elevation—the act of taking control over one’s image in a world where appearance is capital and perfection is never quite attainable.

#### Community-related comparison

A final push factor reported by homosexual participants was the intensified sense of peer comparison and competition within gay male communities. Unlike heterosexual men, whose comparisons often centered on intimate partners, homosexual men described a broader, community-based evaluative gaze, where physical attributes—especially genital size—served as social currency in both sexual and non-sexual contexts.

“You go to the sauna, the beach, even dating apps—it’s always about who’s bigger, leaner, better.” (P11).

“It’s not just about sex. It’s status. Like, people treat you differently if you’re more ‘gifted’.” (P16).

This community-oriented comparison system was shaped by highly visual environments—such as gyms, clubs, sex parties, and digital platforms—where men’s bodies were constantly exposed, evaluated, and ranked. In these settings, penis size was often perceived not merely as a private concern, but as a publicly visible and socially consequential trait.

“Size is not just for your partner—it’s for the guys around you, the way they look or don’t look.” (P19).

“There’s this unspoken hierarchy. You can just feel who’s more confident because of what they have.” (P12).

These perceptions reflect the competitive and appearance-driven norms often found in these environments. While not all participants engaged in such spaces directly, many reported internalizing the standards projected by these environments.

“I don’t even go to those places, but I still feel like I’m being judged by those rules.” (P14).

For some, the pressure extended beyond aesthetics into identity and self-worth, where not having the “right” body—or penis—was associated with feelings of inferiority or social invisibility. PES, in this context, was framed as a tool to gain or restore visibility.

“No one says it out loud, but if you don’t measure up, you’re not really seen.” (P13).

“It’s like, you have to play the game, or you get sidelined.” (P17).

This theme reflects how individuals feel judged by their own community based on idealized physical standards. For many participants, the drive toward PES was not just about attraction or sex, but about feeling included, admired, and validated in peer spaces where physical appearance plays a key role in determining status and attention.

**Figure 2:**
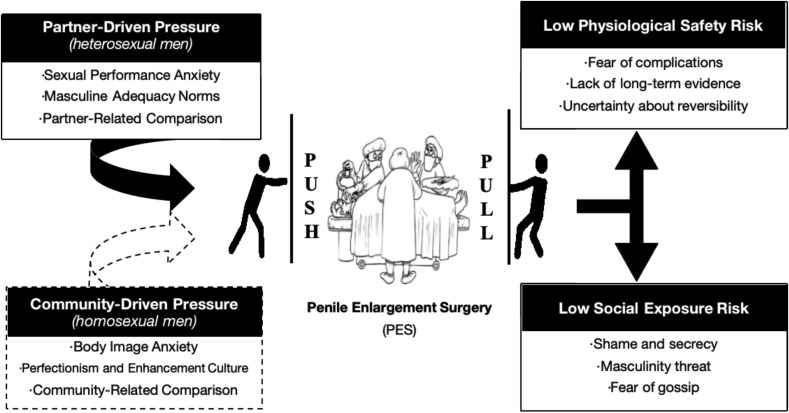
Research model.

This study’s findings, as shown in Figure 2, reveal that while men across sexual orientations share common pull factors to PES, such as fears of medical risk and social exposure, their underlying motivations differ significantly. Heterosexual men’s motivations were largely driven by partner-related pressures, including sexual performance anxiety and concerns about masculine adequacy, while homosexual men’s motivations were more community driven, shaped by body image concerns and social comparisons within peer environments.

## Discussion

This study reveals significant differences in motivations for PES between heterosexual and homosexual men. While both groups share common concerns about medical risks and social exposure, their motivations differ significantly in terms of psychological pathways.

For heterosexual men, motivations for considering PES are primarily driven by partner-related anxiety, particularly sexual performance anxiety and concerns about masculine adequacy. These men often worry about their ability to satisfy their female partners, especially regarding genital size.^23^^,^^24^ Even in the absence of explicit partner criticism, internalized cultural norms lead them to continually doubt their sexual abilities. This performance anxiety often arises from cultural expectations linking sexual performance to male identity. As such, PES is seen as a way to restore confidence and meet perceived relational expectations. For these men, PES is not just about addressing a physical issue but is a response to psychological distress and pressure in sexual relationships.

In contrast, homosexual men are more influenced by community-driven pressures, such as body image dissatisfaction and perfectionist enhancement culture.^25^ Within the gay community, body image is often seen as a key component of attractiveness, particularly in online dating apps, social media, and community interactions where aesthetic standards are emphasized.^26^ Many homosexual men stated that their motivations for PES were not to correct a flaw but to enhance their appearance in line with aesthetic ideals prevalent in their community. These men tend to view PES as part of a broader strategy of self-optimization, rather than simply addressing body dissatisfaction.

The differences in motivations between heterosexual and homosexual men highlight two distinct psychological pathways: relational adequacy in heterosexual men versus visual optimization in homosexual men.^27^^,^^28^ For heterosexual men, PES is largely a private solution to partner insecurity, whereas homosexual men tend to view it as a way to enhance social visibility and desirability within their peer community, emphasizing appearance and body image.

Despite the significant differences in motivations, both groups share similar pull factors, such as concerns about medical risks and social exposure. In particular, the shame and secrecy associated with social exposure were major psychological barriers for many men considering PES. Even when strongly motivated by body dissatisfaction, many men hesitated to pursue PES due to fears of being discovered by others. This highlights the significant role that social evaluation and identity play in PES decisions.

## Limitations

This study, while offering novel insights into the psychosocial motivations behind penile enlargement surgery (PES), has several limitations. The sample was small and demographically narrow, primarily composed of urban, educated individuals, limiting generalizability. The focus on heterosexual and homosexual men excluded other sexual orientations, such as bisexual, pansexual, asexual, and transgender individuals, whose experiences may differ. Additionally, the study focused on intentions rather than actual surgical behaviors, which may vary in real-world conditions. The desire for penile length versus width requires further exploration, as the complications of lengthening may be more severe, introducing complexities in understanding the push and pull factors. Moreover, motivations for PES in homosexual men may differ based on whether they identify as “top” or “bottom”.^29^ Future research should include more diverse sampling and mixed methods to improve generalizability and validity.

## Clinical implications

Clinicians should recognize that motivations for PES are shaped by psychological, relational, and cultural factors—not simply pathology.^30^^,^^31^ Heterosexual men often seek PES due to performance anxiety and fear of partner dissatisfaction, while homosexual men are influenced by body image ideals and community comparison. Pre-surgical assessments should incorporate sexual orientation to tailor counseling approaches: heterosexual clients may benefit from relationship-focused discussions, while homosexual clients may need support around perfectionism and self-worth.^32^ Clinicians should avoid over-pathologizing concerns without considering sociocultural context. Finally, integrated psychological screening and post-procedure follow-up are essential to manage expectations and prevent ongoing dissatisfaction.

## Conclusion

This study reveals significant differences in the motivational pathways toward PES between heterosexual and homosexual men. While both groups share concerns about physiological risks and social exposure, their respective push factors reflect distinct gender roles and body ideals, partner-driven anxiety in heterosexual men versus community-based pressures in homosexual men. These findings underscore the importance of incorporating sexual orientation and psychological context into clinical assessments to provide more tailored counseling and support.

## Supplementary Material

COREQ_Checklist_qfag024
