# Supplementary material for: “Not enough” vs. “never perfect”: a qualitative analysis of penile enlargement surgery intentions in heterosexual and homosexual men
Source: Sex Med. 2026 May 15;14(4):qfag024. doi: 10.1093/sexmed/qfag024 (PMC13178456; doi:10.1093/sexmed/qfag024)
Supplement: COREQ_Checklist_qfag024 [file coreq_checklist_qfag024.pdf]

COREQ (Consolidated Criteria for Reporting Qualitative Research) 32-Item Checklist

Study Title: **“Not Enough” vs. “Never Perfect”: A Qualitative Analysis of Penile Enlargement Surgery Intentions in Heterosexual and Homosexual Men**

Study Type: Qualitative study using semi-structured interviews and thematic analysis

Domain	Item	Guide Questions/Description	Response
**Domain 1: Research Team and Reflexivity**			
Personal characteristics			
1	Interviewer/facilitator	Who conducted the interviews?	Interviews were conducted by the lead researcher (PhD-level), trained in qualitative methods.
2	Credentials	What were the researcher’s credentials?	The lead interviewer holds a PhD in medicine psychology.
3	Occupation	What was their occupation at the time of the study?	At the time of study, the researcher was a master degree in sexual health research.
4	Gender	Was the researcher male or female?	The interviewer identified as male.
5	Experience and training	What experience or training did the researcher have?	The researcher had prior experience conducting sensitive-topic interviews and was trained in reflexive qualitative methods.
Relationship with participants			
6	Relationship established	Was a relationship established prior to study commencement?	No prior relationship was established before recruitment.
7	Participant knowledge of the interviewer	What did participants know about the researcher?	Participants were informed about the researcher’s institutional affiliation, role, and the purpose of the study.
8	Interviewer characteristics	What characteristics were reported about the interviewer/facilitator?	Researcher bias, positionality, and assumptions were discussed within the research team. Reflexivity memos were used.

**Domain 2: Study Design**			
Theoretical framework			
Participant selection			
9	Methodological orientation	What methodological orientation was stated to underpin the study?	Constructivist grounded theory informed the analysis. The Push-Pull model was the guiding framework.
10	Sampling	How were participants selected?	Purposive sampling based on sexual orientation and PES experience.
11	Method of approach	How were participants approached?	Recruitment was done via online forums and messaging. Screening was conducted online.
12	Sample size	How many participants were in the study?	24 participants (12 heterosexual, 12 homosexual).
13	Non-participation	How many people refused or dropped out? Why?	26 individuals were excluded due to criteria mismatch or withdrawal.
Setting			
14	Setting of data collection	Where was the data collected?	All interviews were conducted via encrypted video or audio calls.
15	Presence of non-participants	Was anyone else present besides the participants and researchers?	Only the participant and interviewer were present.
16	Description of sample	What are the important characteristics of the sample?	Detailed in Table 1 (age, education, sexual orientation, occupation).
Data collection			
17	Interview guide	Was the interview guide provided? Was it pilot tested?	A semi-structured guide was developed and pilot-tested.
18	Repeat interviews	Were repeat interviews carried out?	No repeat interviews were conducted.
19	Audio/visual recording	Did the research use audio or visual recording?	Interviews were audio-recorded with consent.
20	Field notes	Were field notes made during and/or after the interview?	Field notes were taken after each interview.
21	Duration	What was the duration of the interviews?	Interviews lasted 60–90 minutes.
22	Data saturation	Was data saturation discussed?	Thematic saturation was reached after 22 interviews.

23	Transcripts returned	Were transcripts returned to participants?	Transcripts were not returned, but 5 participants reviewed summaries (member checking).
**Domain 3: Analysis and Findings**			
Data analysis			
24	Number of data coders	How many data coders were involved?	Two researchers independently coded all transcripts.
25	Description of coding tree	Did authors provide a description of the coding tree?	A coding structure with themes/subthemes was developed and shared in the manuscript.
26	Derivation of themes	Were themes identified in advance or derived from the data?	Themes were derived inductively from the data.
27	Software	What software was used to manage the data?	NVivo 14 was used for coding and data management.
28	Participant checking	Did participants provide feedback on the findings?	5 participants reviewed summaries for accuracy.
Reporting			
29	Quotations presented	Were participant quotations presented to illustrate the themes?	Participant quotes are included to support all key themes.
30	Data and findings consistent	Was there consistency between the data presented and the findings?	Yes; findings are well supported by participant narratives.
31	Clarity of major themes	Were major themes clearly presented?	All major themes are clearly outlined in the results section.
32	Clarity of minor themes	Is there a description of diverse or minor themes?	Minor or divergent cases are briefly discussed in analysis.
